# Provocation test in patients with different phenotypes of aspirin hypersensitivity

**DOI:** 10.1186/2045-7022-3-S1-P8

**Published:** 2013-05-03

**Authors:** Krzysztof Specjalski, Lucyna Gorska, Ewa Jassem

**Affiliations:** 1 Medical University of Gdansk, Poland; 2 Medical University of Gdansk, Department of Allergology, Poland

## Background

Aspirin (ASA) hypersensitivity has a complex clinical presentation with three major phenotypes: aspirin-exacerbated respiratory disease (AERD; asthma and/or nasal polyps), chronic urticaria and aspirin-induced anaphylaxis. In patients with AERD asthma is often severe and difficult to control. Moreover, NSAID ingestion may provoke life-threatening attack. Thus, diagnosing of ASA hypersensitivity is an important issue. In everyday practice ASA hypersensitivity is confirmed by oral (OPT), nasal (NPT) or bronchial provocation tests. However their protocols vary in terms of maximum cumulative dose. Besides, phenotype of hypersensitivity is not taken into consideration when choosing the protocol.

## Aim of the study

The aim of this study was to compare responsiveness to ASA in patients with different phenotypes of ASA hypersensitivity (AERD, chronic urticaria, anaphylaxis).

## Patients and methods

238 patients (179 women, 59 men) aged 19-71 with the history suggesting hypersensitivity to NSAIDS were provoked with aspirin. The group comprised 92 patients with AERD, 83 with chronic urticaria and 63 with suspicion of anaphylaxis caused by ASA. Single-blind oral provocation test was performed according to two-day protocol (day 1- placebo, day 2- increasing doses of ASA: 50, 100, 150, 300, 400 mg). Anterior rhinomanometry, spirometry and clinical monitoring were used to assess the reaction. The test was considered positive in case of clinical symptoms of hypersensitivity, significant decline of FEV1 or positive nasal response in anterior rhinomanometry.

## Results

Provocation test was positive in 81 (34%) patients, including 26 with chronic urticaria, 45 with asthma and/or nasal polyps and 10 with history of anaphylaxis. Proportions of positive results in the subgroups were 31%, 49% and 16%, respectively. The mean dose provoking reaction was significantly lower in AERD group compared to patients with chronic urticaria (212 mg vs. 604 mg, p<0.05). 36% of positive responders with asthma reacted to doses ¡Ü200 mg. 19 subjects (8%) responded to placebo making the provocation impossible to evaluate.

## Conclusions

Patients with AERD usually respond to smaller doses of ASA compared to patients with chronic urticaria. As an oral provocation test is associated with the risk of anaphylactic reaction and its severity is dose-dependent it may be suggested to introduce new protocol for patients with severe asthma, based on lower first doses of ASA.

